# Constructing Imperial Authority: The Intersection of British Imperial Constitutional Law and Private International Law

**DOI:** 10.1093/ojls/gqag012

**Published:** 2026-02-28

**Authors:** Roxana Banu

**Keywords:** imperial constitutional law, imperial authority, private international law, conflict of laws, AV Dicey

## Abstract

Historians and constitutional law scholars are starting to uncover the imperial dimensions of the British constitution. But our accounts of the nature of authority in the British imperial context remain incomplete without an engagement with private international law, which played a significant role in conceptualising imperial authority. This article focuses on the forgotten interplay between imperial constitutional law and private international law. It shows how key doctrinal principles of private international law were referenced either as alternatives to or counterparts of key imperial constitutional law principles. Imperial actors would appeal to one or another image of imperial authority constructed by either imperial constitutional law or private international law to gain more autonomy or to tighten control. Far from being a relic of the past, the significance of this history can be traced in contemporary cases and debates about the nature of authority in the UK and its overseas territories.

## 1. Introduction

Imperial authority in the British Empire was constructed as an unequal distribution of local autonomy and control from the metropole.^1^ Historians of empire have provided enormously enriching accounts of the ‘jurisdictional jockeying’ that occurred, as different actors navigated the dizzying maze of multiple sites of authority available throughout the empire.^2^ While constitutional law scholars in former colonies have always engaged with the imperial dimensions of constitutional law, the last few years have also seen a renewed interest in problematising an imperial perspective on the British constitution.^3^ The emerging picture of legal authority in the imperial context is a mix of both liberty and control,^4^ of ‘constitutional autonomy for the white settler colonies’ and varying degrees of ‘paternalist despotism’ and indirect rule for the rest of the empire.^5^

But this melange of unequally distributed patterns of authority depended on the principles and doctrines of another field, which has remained remarkably silent about its imperial past: private international law (or conflict of laws). Unlike in constitutional law, there is no account of the colonial history of private international law,^6^ so that constitutional law and private international law scholars remain unaware of the imperial entanglements of the two fields.^7^ Yet, in the context of the British Empire, imperial constitutional law and private international law operated in tandem to operationalise a ‘hopeless prospect of having ultimately to reconcile the unity of the whole with the autonomy of the constituent parts’.^8^ Moreover, the intersection was framed by scholars who were trained in and often taught both fields. This allowed them to position arguments for more local autonomy or stronger metropolitan control by appealing to arguments and principles of both fields in different contexts. Looking back, the imperial history of both fields is so deeply entangled that it would be difficult to dissociate them in any meaningful way.

Acknowledging this joint imperial history not only helps us make sense of the nature of authority in the British imperial context; it is also helpful in making sense of contemporary cases that seem to bring old imperial dilemmas into our own legal context. Several recent cases have raised questions about the administrative and legislative authority of the UK for British Overseas Territories. These cases reveal a significant ambivalence about the nature of authority. For some purposes, a legal pluralist scene is posited. We are told, for example, that the governor of Diego Garcia, responsible for the British Indian Ocean Territory but operating from London, can be sued in his individual capacity in England, but that his public law duties must be examined in the courts of the distinct jurisdiction of the British Overseas Territories.^9^ Legislative enactments for the Chagos Islands cannot be challenged on the basis of Magna Carta principles or human rights norms unless they have been extended specifically to those ‘separate’ jurisdictions.^10^ For other purposes, we must imagine an indivisible realm. A decision to exile the population of the Chagos Islands can be made in the name of the security interests of the whole realm; expulsions or restrictive immigration and asylum policies in one part of the realm can be justified by reference to the interests of another part or of the whole.^11^

Read from the vantage point of constitutional law only, these cases are puzzling.^12^ But, as I show in this article, the nature of imperial authority has always been conceptualised at a porous meeting point between imperial constitutional law and private international law. The ambivalence of conceptualising different parts of the empire as distinct jurisdictions, as well as indivisible parts of a whole, was crafted at the intersection of these two fields.

To highlight some of the dimensions of this history, in this article I take as a starting point the debates occurring in the white settler dominions in the first three decades of the 20th century. It is in these three decades that dominions capitalised on the vocabulary of private international law which sustained a legal pluralist scene throughout the empire.^13^ It enabled them to argue that while they could not be states under imperial constitutional law or public international law, they were recognised as states under private international law. That perspective could then be fed back into and connected with constitutional law doctrine, so that dominions could move from legal autonomy to political independence. Because at least by the late 19th century private international law was viewed as an independent legal field, it could offer alternative conceptual and analytical elements of its own to counterbalance imperial constitutional law ones. But the arguments in the first three decades of the 20th century relied on older 19^th^ century cases, when the borderline between imperial constitutional law and private international law was much more difficult to discern.^14^ Therefore, imperial actors could maintain an ambivalence between portraying private international law concepts as alternatives to or counterparts of imperial constitutional law ones in different contexts for different purposes.

I begin, in section 2, by offering an overview of the two versions of imperial authority that were framed at the intersection of private international law and imperial constitutional law. I show that the nature of imperial authority was conceptualised in its inward looking, top-down facet by imperial constitutional law and in an outward-looking, bottom-up fashion by private international law.^15^ What is more, this dual conceptual frame was shaped, perhaps unwittingly, by the writings of the two main British imperial constitutionalists and private international law scholars of the late 19th- and early 20th century, Albert Venn Dicey and Arthur Berriedale Keith.

In section 3, I outline the private international law doctrine that sustained this bottom-up framing of authority for the dominions and its imperial ramifications. Private international law allowed for two shifts which would not have been available in constitutional law (or even public international law). First, private international law’s theories of territoriality allowed dominions to argue that they had plenary authority to regulate matters of concern to the dominions, even with extraterritorial effect, while acknowledging that other parts of the empire (including the metropole) were free to refuse to enforce this extraterritorial reach of authority. Second, some scholars have used the private international law framework to argue that the metropole itself was a local unit, rather than a central constitutional organ for the empire as a whole.

In section 4, I take stock of the consequences of moving back and forth between two conceptualisations of imperial authority. I show that legal pluralism throughout the empire could simultaneously be condoned and rejected, depending on the legal and political domain in which it was used and on the economic interests of the empire.

Finally, I conclude in section 5 by unpacking the value of recovering the imperial intersection of private international law and constitutional law in our contemporary context. This history not only allows us to make sense of contemporary cases involving British Overseas Territories, but also offers us a different perspective on the nature of authority in the British postimperial realm.

## 2. Janus-Faced Imperial Authority

It is well known that authority in the British Empire was not premised on a broad principle of assimilation. Instead, different parts of the Empire enjoyed different levels of legislative and adjudicatory autonomy and different governance structures, often shifting over time. At the turn of the 19th century, for example, the colonial office classified the colonies according to the nature and distribution of their local legislative powers and the respective calibration with control from the Crown.^16^ Furthest on the spectrum of legal autonomy were those dominions that possessed elected assemblies and responsible governments, and the Crown retained a veto on legislation.^17^ Throughout the first half of the 20th century, the dominions of the Union of South Africa, Canada, Australia, New Zealand and later the Irish Free State moved further towards legislative autonomy with a respective gradual diminishing control or veto power from the Crown.

There was also a dizzying array of law sources for different parts of the empire.^18^ The starting premise was that ‘if there be a new and uninhabited country found out by the English subjects, as the law is the birthright of English subjects, so wherever they go they carry their laws with them’, although ‘only so much of the English law as is applicable to their own situation and the condition of an infant colony’.^19^ In conquered colonies, the existing law remained in place until and insofar as it was altered by the conqueror.^20^ Special provisions were often made for the observance of ‘native’ or ‘customary’ laws.^21^ But the starting points were deceptively simple. There was ample controversy as to the precise date on which English law was imported in a particular colony; the scope of the law imported was unclear, since English ‘laws originating out of a purely local policy’ were not meant to be ‘exported’;^22^ the methods of ascertaining customary laws and their validity,^23^ and the extent to which they were to be protected when representative government was granted to the dominions, were constantly in debate.^24^ And, of course, designating a place as uninhabited was a highly manipulative project,^25^ as was the distinction between settled and conquered colonies.^26^

Sitting at a higher level of abstraction from this immense legislative complexity of the British Empire were a cluster of principles meant to *calibrate* and *conceptualise* the level of local autonomy and metropolitan oversight for different parts of the empire and sometimes in different domains (shipping versus criminal law versus private law). *Calibrating* this tension was done primarily through two main doctrines—limiting the authority of legislation passed in the colony to the territory of the colony (the doctrine of territorial limitation) and invalidating legislation or customary laws that were ‘repugnant to the law of England’ (the repugnancy doctrine).^27^ As colonies pushed to increase their autonomy, both doctrines were softened. The repugnancy doctrine was limited in 1865, when the Colonial Laws Validity Act clarified that laws passed in the colonies would be held repugnant only if they contravened specific imperial statutes which applied to the colony, rather than contravening broader rule-of-law principles of English law.^28^ It took much longer to dismantle the doctrine of territorial limitation through a legislative enactment. It was eliminated by the Statute of Westminster in 1931 for the dominions only, remaining in place for the rest of the British Empire.^29^

*Conceptualising* legal authority in the British Empire was a different matter altogether. There was no attempt from England to provide a grand theory of autonomy and/or legal authority in the empire.^30^ The hope was that a case-by-case application of the doctrines referenced above would clarify the scope of authority in a granular and pragmatic fashion. But this was a challenging proposition. AV Dicey wrote in 1886 that ‘among the political arrangements devised by the ingenuity of statesmen none can be found more singular, more complicated, or more anomalous than the position of combined independence and subordination’.^31^

If a theory had to be devised, in constitutional law terms, the conventional view from the metropole—expressed by Dicey and Arthur Berriedale Keith, the two main imperial constitutional law scholars of the late 19th century to the interwar period—was that colonial legislatures were non-sovereign legislative bodies because their action was restricted by laws that they could not change, given the two doctrines of imperial control mentioned above.^32^ But while subordinate to the Westminster Parliament, colonial legislatures were not delegates of the imperial legislature, according to a series of Privy Council decisions from 1878 to 1885; they were legislatures restricted in the area of their powers, but within the area of their competence they were supposed to be unrestricted and not acting as an agent or a delegate.^33^

From this followed a few conclusions which—at least until the separate signatures of the Treaty of Versailles by the Dominion of Canada, the Commonwealth of Australia, the Union of South Africa, the Dominion of New Zealand and India—seemed to stand firm from a constitutional law and public international law perspective. Colonies could not be viewed as separate states either under imperial constitutional law or under international law.^34^ They were ‘mere geographical expressions’ of imperial government.^35^ Furthermore, there was no separate nationality associated with any part of the empire.^36^ Consequently, defining the territorial reach of colonial legislation was often difficult. If colonies were not subjects of international law, it was unclear whether the three-mile limit rule for defining territorial waters would apply to their territory,^37^ and if they had no subjects but had legislative competence for the ‘peace, order and good government’ of the colony, it was unclear whether they could legislate for the activities of their inhabitants abroad in matters that touched on the welfare of the colony.^38^ Similarly, the mere boundaries of colonies were disputed at times, but it was difficult to conceptualise the possibility of colonies suing each other to define the territorial reach of their duties.^39^ Nor was it easy to understand how and whether disputes over ownership of colonial land could be allowed between different colonies. Since they were neither corporations nor states, it appeared, in Thomas Baty’s characteristically witty formulation, ‘like the suit of the cook by the butler because too much was spent on coals, or the suit of the War Office by the Board of Agriculture because men who might be soldiers were kept to work at the harvest’.^40^

But there was also a different way of framing the structure of the empire and the nature of imperial authority coming from private international law. This area of the law was meant precisely to manage legal pluralism. It would be tasked with clarifying which court has jurisdiction to hear a dispute connected to multiple legal systems; which law applies to regulate the rights and liabilities of the parties; and whether a foreign judgment could be recognised and enforced abroad. Throughout the 19th century, the height of imperialism, this body of law knew its greatest development in virtually all imperial metropoles.^41^ The field was primarily meant to settle conflicts of laws between imperial metropoles, viewed as independent sovereigns of equal ‘civilisation’. However, in the 19th century in the British Empire, private international law was deeply embedded in the broader framework of colonial law and the management of legal pluralism in the imperial context.^42^ What remains unacknowledged is that in the imperial context, at least in the later part of the 19th century, private international law provided not only a framework for managing conflicts between jurisdictions, but a vocabulary for defining what amounted to separate jurisdictions in the first place.

In England, the main architect of private international law, as well as constitutional law, in the second half of the 19th century until 1922 was Albert Venn Dicey. By the start of the 20th century, Dicey had become interested in the intersection of the two fields in an imperial context, taking the question of the validity of laws passed by colonial legislations as a point of departure when re-editing both his conflict of laws digest and his treaties on constitutional law. It is therefore not surprising that Dicey entrusted the editorship of his conflict of laws digest to Arthur Berriedale Keith, the main imperial constitutional law scholar of the interwar period.^43^

Dicey’s scholarship in both private international law and constitutional law is well studied.^44^ Yet, there is little engagement with Dicey’s work across the two disciplines.^45^ Similarly, while Keith was undoubtedly recognised as a renowned imperial constitutional law scholar and Indologist, he does not register in the historical memory of private international law. Furthermore, the correspondence between Dicey and Keith has never been studied as an engagement across disciplines on the nature of authority in the British Empire.^46^

This has left a blind spot. For example, Dylan Lino, in his helpful setting of Dicey’s constitutional law writings—but not his private international law writings—in an imperial context,^47^ found it puzzling that Dicey never had a clear answer about the territorial reach of the imperial constitution or that he had no answer for how abuse of power could be reined in throughout the empire. In fact, why Dicey could simultaneously accept imperial sovereignty and legal pluralism throughout the empire remains a puzzle. What is more, within the entire constitutional structure of the British Empire, Dicey offered no theory of the state *per se*, no pondering on how to define the country or many countries of the British Empire, if that is what they were.

It may thus come as a surprise that we get a first glimpse of the particular way in which private international law conceptualised imperial authority precisely from Dicey’s definitions of the term ‘country’ in his book on domicile in 1879,^48^ which then reappeared in the various editions of his *Digest on Conflict of Laws* from 1896 onwards.^49^ In discussing the ascertainment of one’s domicile in ‘a country’ or the cross-border recognition of a right vested in ‘a country’, Dicey invited us to distinguish between the term in a geographical, historical, political and legal sense. A country in the political sense of the word was ‘the whole of the district or territory subject to one sovereign power … such as the British Empire’.^50^ Within this political ‘realm’, the power of the sovereign over his territory and his subjects is supreme.^51^ In a geographical sense, it was ‘a geographical district making up a separate part of the physical world … as in a newly discovered country’.^52^ In its historical sense, ‘it means a land inhabited, or supposed to be inhabited, by one race or people as for instance Italy, before the Italians were united under one government’.^53^ Dicey concluded that ‘neither the geographical nor the historical sense of the word concern writers on law’, and that the political sense is not useful for private international law. Therefore, only the legal sense matters, namely ‘a district or territory, which (whether it constitutes the whole or a part only of the territory subject to one sovereignty) is the whole of the territory subject to one system of law’.^54^ So, while the British Empire was one country in the political sense of the word, it ‘consists of a large number of countries in a legal sense’. Furthermore, via a fiction, they were to be considered ‘foreign’ in relationship to each other and to the metropole.^55^ In private international law, this proposition seems to have been obvious, so that Dicey did not feel the need to include widespread citations to support it.^56^

The fiction may appear ‘thin’,^57^ and Dicey recognised it may even be offensive.^58^ But it was also quintessentially British, or at least particular to the common law tradition of private international law. In continental Europe, private international law was viewed as a branch of international law more broadly and, like public international law, was one that was meant to coordinate authority between equal sovereign states.^59^ The notion of territoriality—and its twin notion of personality—were simply two different analytical prongs through which to conceptualise a universal distribution of state authority. However one sliced the pie of authority along the territorial and personal dimensions, the framework would only get underway once sovereign states of equal levels of ‘civilisation’ were in place.^60^

By contrast, the fiction of separate legal districts akin to foreign states *inter se* was the result of particular English aspects of private international law history and political thought. For one thing, the historical roots of English private international law lay in notions of territorial authority specific to the feudal legal system. For questions of jurisdiction and then choice of law, a delineation was thus always made based on a principle of territoriality.^61^ Furthermore, even throughout the 19th century, English private international law continued to decouple notions of territoriality from state sovereignty. Dicey, who was notoriously sceptical of the fiction of the state, refused to conceptualise the role of the field as determining a universal distribution of authority among states in a political sense. Equally implausible was adopting the analogy between private and public international law prevalent on the continent. Instead, Dicey argued that private international law was meant to secure the extraterritorial recognition of vested rights. To continental European scholars, this definition was circular, since it lacked an explanation of which state had authority to vest rights. For Dicey, this was partly beside the point, since the notion of territoriality could explain much of the conundrum and, for him, rights were at least to some extent pre-institutional.^62^ Ironically, Dicey could reject importing a political notion of the state from constitutional law or public international law, by embracing a different, ‘legal’ sense of the state in private international law, which better coupled with his views on the nature of rights and the common law.

In the British Empire, therefore, the fiction of ‘territoriality’ could sustain, via private international law, the fiction that colonies were akin to foreign states *inter se*.^63^ As Canadian lawyer Emerson Read explained in a 1935 book widely read in the dominions, under Dicey’s theory, all that was needed for a colony to be seen as a foreign country under private international law was for it to function as ‘a unit of territory which has a single body of law distinct from that of the territory of the forum’.^64^ ‘The courts, *however constituted*, and the precepts, *whatever their source*, applied by them in a unit of territory constitute the courts and law of that territory and of no other. Law is not personal in its application, but territorial.’^65^

A few consequences flowed from the particular understanding of imperial authority via private international law as opposed to imperial constitutional law. First, under imperial constitutional law, a state was to be understood in its political sense and was synonymous with sovereignty, whereas under private international law, statehood was to be understood via the legal concept of territoriality. For example, in 1912, the *Canadian Law Times* published an article in which the question was raised whether corporations formed under colonial legislation—referred to as ‘foreign … constantly in reports and textbooks’—could operate extraterritorially.^66^ If colonies were territories of the empire without sovereignty, their legal creations (including corporations) could not operate extraterritorially. If they were states in a political sense, they could enter into reciprocal treaties for the recognition of the status of their corporations. Private international law offered an intermediate route—colonies did not have to be sovereign in a political sense for their respective laws and corporations to be recognised abroad via comity.

Second, while in constitutional law or international law terms it was extremely difficult to conceptualise suits between different parts of the British Empire, it was possible to argue that since ‘territorially they are mutually exclusive’, they could sue each other and ‘the conflicts of their laws are those which belong to private international law’, with all the perplexing questions resulting from this proposition.^67^

Third, under imperial constitutional law, there was only one empire-wide citizenship. Yet, once naturalisation was left to the competence of the different units as ‘foreign countries’, a person could be a British citizen in one part and an alien in another, because naturalisation laws were considered limited to the territory of each dominion. However, in 1902, New Zealand’s Attorney General John Salmond argued for a private international law perspective of ‘local side-by-side with imperial citizenship’.^68^ In his view, the status of a British subject could only be granted by imperial authority, rather than by the patchwork system of naturalisation laws, but domicile^69^ could create a type of local citizenship which dominions could regulate and protect. By 1913, Canadian scholar AHF Lefroy could write that the ‘“expression subject of a colony” has high judicial authority and perhaps, may be taken to mean British subjects domiciled in the colony’ so that ‘it seems beyond question that [dominions] must have the same power to bind their own subjects everywhere, as the Imperial parliament has to bind British subjects everywhere’.^70^

## 3. Imperial Ramifications

It might appear straightforward enough to suggest that the legislatures were supreme within the limits granted by the metropole. But, as Dicey admitted, this was not so much a theory of imperial authority as ‘a political arrangement’ which was ‘kept in tolerable working order by a series of understandings and of mutual concessions’.^71^ It relied on the ‘cheerful acquiescence’ by the colonies of the imperial bond, an acceptance that local self-government ‘does not constitute a nation’ and a certain degree of local prosperity.^72^ It was always inherently unstable, but by the turn of the 20th century the prerequisites were starting to falter even for the self-governing dominions. And when they did, the language of private international law offered an alternative conceptualisation of independence which could creep into imperial debates without seeming revolutionary or unfamiliar to the metropole, though it sometimes caused significant confusion.^73^

In this section, I argue that private international law allowed for two moves which would have been unavailable in either public international law^74^ or imperial constitutional law: first, conceptualising the issue of extraterritoriality as a question of cross-border *recognition* of the limits of authority, rather than a question of the *existence* of authority in any part of the empire;^75^ and second, the provincialisation of metropolitan legal authority. These arguments produced an unequal distribution of legislative authority throughout the empire. They allowed dominions a higher level of legislative autonomy, but at the expense of weaker colonies and of already marginalised British subjects, particularly people of colour. Furthermore, while these arguments were crafted by the dominions as tools of self-empowerment, they also enabled the dominions and the metropole to transform them into shields from responsibility for colonial abuses in various parts of the empire.

### A. Reconceptualising the Doctrine of Extraterritoriality

Under imperial constitutional law, the authority of a colonial legislature was limited to the territory of the colony or dominion. The exact origin of this limitation was not always clear.^76^ It was sometimes described as a limitation inherent in the nature of colonial legislatures.^77^ Alternatively, its roots were traced to a *dictum* expressed in *Macleod v New South Wales*.^78^ McLeod was convicted of bigamy in New South Wales under a colonial statute providing that ‘whosoever being married marries another person during the life of a former husband or wife, wheresoever such second marriage takes place, shall be liable to penal servitude for seven years’. This seemed to imply an extraterritorial authority to punish bigamy, wherever it may occur. It was broadly conceded that the statute was poorly drafted because it failed to clarify, as the English variant of the bigamy statute did, that it only applied to British subjects.^79^ But in *obiter dicta*, the Privy Council also noted that the jurisdiction of a colony ‘is confined within their own territories, and the maxim which has been more than once quoted, “*Extra territorium jus dicenti impune non paretur*”, would be applicable to such a case’.^80^

Several other cases on a broad range of legal matters relied on the principle of the territorial limitation of dominion legislation. It was considered a violation of the principle of territorial limitation for dominions to: divorce people domiciled in England or Scotland (even if long-term residents in the colonies);^81^ extend an Australian statute declaring certain acts as not amounting to libel to publications spread across Australia and England;^82^ or collect taxes under an Australian act for companies registered in England despite significant commercial and investment activity in Australia.^83^ It was understood that, in theory, this limitation applied to an ‘unlimited range’ of issues, including ‘taxation, shipping, air navigation, marriage, criminal law, deportation and the enforcement of laws against smuggling and unlawful immigration’.^84^

This limitation was abolished by the Statute of Westminster of 1931 in the case of autonomous dominions,^85^ but it continued for other parts of the empire.^86^ The three decades of the 20th century leading up to the Statute of Westminster were marked by a shift in register, whereby scholars and colonial officers in the dominions started to rely on private international law doctrine to slowly carve a path to legislative independence from the metropole. Private international law became a useful vocabulary to articulate the view that dominions, because they were designated as akin to foreign countries in relationship to each other and the metropole, could not be limited in their authority by any other part of the empire, although their extraterritorial reach could be refused. One important part of this shift lay in convincing the metropole that the doctrine of extraterritorial limitation resulted from a failure to appreciate the meaning of this concept in private international law, as opposed to imperial constitutional law. This argument travelled at remarkable speed throughout the dominions.^87^

In Australia, the argument was first put forward in 1900 by W Harrison Moore, Professor of Law at the University of Melbourne, who would later represent Australia at the League of Nations.^88^ Moore argued that while the importance of distinguishing between the operation of laws in the empire via imperial constitutional law or via private international law was ‘obvious’, it was often ignored.^89^ Some laws were ‘in operation in the colony’ by virtue of imperial legislation covering the entire empire or clear prerogative authority from the metropole and some laws were ‘in operation in England in respect of the colony’ so that ‘any recognition they may obtain in the Colonies is due, not to any paramount jurisdiction, but to the “comity of nations” or whatever principle underlies the “Conflict of Laws”’.^90^ This confusion mattered. Cases which led to the prevailing, though ‘not universal’, view that the territorial limitation on colonial legislatures was a rule in restraint of power (rather than merely a rule of interpretation which applied to the law of any sovereign state) were in fact ‘decisions not of Courts of the colony whose power is in question, but of an English Court or the Court of another colony, asked to recognise and give effect to the law on grounds of comity’.^91^

The same argument was reiterated in Canada. AHF Lefroy, a highly regarded constitutional law scholar at the University of Toronto, published *Canada’s Federal System; Being a Treatise on Canadian Constitutional Law Under the British North America Act* in 1913, in which he argued that it was

a moot question whether colonial statutes, purporting to have an extra-territorial operation, are, nevertheless, not valid and binding within the territory and upon the courts of the lawmaker … it being quite a different question whether foreign Courts will recognise them and judgments obtained in legal proceedings initiated under them.^92^

His earlier book, *The Law of Legislative Power in Canada*,^93^ had already discussed a series of cases which were supposed to ‘bring prominently into notice the distinction already referred to, between the question whether such statutes are valid and binding within the territory and upon the Courts of the law-maker, and the question whether foreign courts will recognize them’.^94^

A widely read book titled *The Canadian Constitution*^95^ was published in 1918 by Walter S Scott, one of the inaugural lecturers at the Faculty of Law of the University of Alberta in Edmonton and Counsel to the Legislative Assembly of Alberta.^96^ Scott included four principles of the concept of extraterritoriality meant to distinguish between the imperial constitutional law and the private international law dimensions of the concept. He argued that in the case of imperial law, ‘judges of the English courts have never to think of whether the legislation is *ultra vires* or not. It cannot be *ultra vires*’, yet ‘imperial legislation may be disregarded in the courts of other countries as opposed to the comity of nations, or, in other words, contrary to private international law’.^97^ So, too, dominion or provincial legislation passed by ‘legislatures restricted in the area of their powers, but within that area unrestricted, and not acting as agents or delegates of the imperial legislature’, ‘though *intra vires*, might, like Imperial legislation, be disregarded in other jurisdictions, as being contrary to the principles of private international law’.^98^

Given his expertise in private international law, public international law^99^ and imperial constitutional law, Arthur Berriedale Keith intervened in the debates. He acknowledged that private international law cases were constantly referenced in discussions on the doctrine of territorial limitation under imperial constitutional law, but he thought this had only given rise to confusion and that law officers might have provided false hopes for greater autonomy by mixing private international law and imperial constitutional considerations.^100^ In his view, if a colony can levy estate duties on the whole of the personal property, wherever situated, of a person who died domiciled in the colony, it is not because it possesses extraterritorial competence under imperial constitutional law, but because of the private international law connecting factor of ‘the situs of goods’.^101^ But Keith knew very well that the distinction was unstable. Different ways of ‘locating’ goods implied a broader or narrower extraterritorial competence. The imperial government argued that the ‘situs’ of shares is the place where the share is registered or the place where it is transferred, while the Cape and Transvaal localised shares at the place where the company exercises its operations. It was thus possible for the colonies to argue that the metropole was trying to tax ‘their corporations’ ‘extraterritorially’, while the metropole could argue that the colonies were attempting to exercise extraterritorial authority over its ‘domiciliaries’. There were only two ways out of this impasse: either admitting that England’s authority extended over the entire empire, and therefore took precedence over that of the colonies, or that if coordinating choice of law rules was not possible, the colonies and the metropole could reject each other’s extension of authority, leading to the classical ‘limping relationships’ problem under private international law. People could be seen as divorced in India but still married in England,^102^ and death duties could be applied in one way in Transvaal and in another way in England, leading to serious economic hurdles.^103^ For unionists like Dicey and Keith, the latter proposition seemed implausible,^104^ but for the dominions it was a significant way of pushing for greater autonomy and legislative authority. For this reason, Keith’s contention that private international law arguments should be excluded from discussions of extraterritorial authority under imperial constitutional law fell on deaf ears.

The debates culminated in a highly influential article published in 1917 by Sir John Salmond, New Zealand’s Solicitor-General at the time, scholar of jurisprudence and constitutional law, who taught private international law at Adelaide University. Salmond sought to press ‘an essential distinction between making laws in one place to have effect in another, and making laws in one place to have effect in that place with respect to things done in another’.^105^ The latter was ‘a question of the constitutional limitations of the legislative authority of the New Zealand Parliament’, while the former was ‘a question of private international law’ relating to the cross-border recognition of the extraterritorial effect of a local law.^106^ Westminster had allegedly confused the two matters and assumed that dominions were claiming an absolute right to have their laws with extraterritorial operation *recognised* in other parts of the empire and beyond, when in fact they only claimed the *authority* to pass laws with extraterritorial effect for enforcement within their own territory. Viewed from the metropole, the two issues were easily conflated: Westminster had to *recognise the authority* of a colony to pass a law with extraterritorial effect. By contrast, Salmond was suggesting that the authority to pass such legislation was inherent in the powers of government of a colony, as much as it was enshrined in the powers of government of the metropole.^107^ Westminster only had the authority, under private international law, to decide if it would *recognise the extraterritorial effect* of a colonial legislation if it purported to operate in England. Salmond, otherwise a keen imperialist, was in effect holding the British Empire to the legal pluralist scene it purported to create. This, he thought, implied that different parts of the empire engaged with one another equally under principles of private international law.

As Canadian scholar Herbert Smith remarked, the extent to which dominions would be minded to pass laws with extraterritorial effect would be informed by notions of ‘comity’ and ‘international law’, rather than any imperial constitutional law restrictions.^108^

The theory that Dominion legislatures are subject to some vague territorial limitation from which the British Parliament is free is in reality part of the old idea, never precisely defined, that they are in some sense imperfect legislatures of an inferior type.^109^

### B. Provincialising Imperial Authority

Salmond did not go so far as to suggest that the reach of authority was equal for the metropole and the colonies. He admitted that ‘the *territorium* of the Imperial Parliament is the whole Empire and not merely the United Kingdom, whereas the *territorium* of a colonial legislature is limited by the boundaries of the colony’.^110^ For Salmond, there was no question that imperial legislation could operate throughout the empire whereas laws passed in the colonies operated in other parts of the empire only via comity or other principles of private international law. But others, relying on private international law, sought to go much further, in effect provincialising metropolitan authority, by arguing that England’s authority was also *prima facie* limited to the territory of England.

In an article published in 1908, Canadian lawyer CC McCaul brought Dicey’s statements to their logical conclusion when challenging the perception at the time that

as against British subjects resident in other portions of the King’s dominions, &c., English jurisprudence holds that the service of the King’s writ issued by the English court is ‘effective’, while the service of the King’s writ issued by the Colonial court is ‘ineffective’.^111^

This was precisely a question pertaining simultaneously to imperial constitutional law and private international law. Rules for service out of the jurisdiction were a quintessential aspect of private international law principles on jurisdiction, but they implied an element of extraterritorial authority. From the perspective of imperial constitutional law, colonies lacked precisely this extraterritorial authority.^112^

McCaul argued that this pitting of imperial authority against territorially limited authority in the colonies was entirely mistaken: a default judgment pronounced in England

is purely an *English* judgment, not a British judgment, much less an Imperial judgment. The judgment is a ‘foreign’ judgment just as much in the Colonies as in France or Germany: execution of the judgment is ‘ineffective’ beyond the limits of its territorial area—England.^113^

McCaul in effect turned the propositions made within private international law about the colonies as ‘foreign states’ against imperial authority. As ‘foreign countries’, laws enacted and judgments rendered in the colonies and the UK operate in relationship to each other according to ‘principles of international law’—by which McCaul, as the vast majority of actors making such references, meant *private* international law, as colonies had no recognised status under *public* international law at the time of his writing. Therefore, every statute passed either in the colony or in the metropole would be subject to some kind of unspecified limitation—based on international effectiveness—of territorial reach. But if this is so, it meant that no statute passed in the metropole—including one which seemed to allow default judgments against British subjects throughout the empire—could be viewed as having an Empire-wide application. Instead, a private international law interpretation suggested two possibilities. On the one hand, the statute could be viewed as ‘municipal’, rather than ‘imperial’, legislation. According to McCaul, it must be that

Parliament, aware that the Courts have determined that a Colonial judgment is a ‘foreign’ judgment in England, and an English judgment a ‘foreign’ judgment in the Colonies, has left the international (which *pro hac vice* includes the Colonial) aspect of the question severely alone.^114^

On the other hand, perhaps the jurisdiction to issue a default judgment was based on the fact that it meant to bind ‘a subject of the sovereignty of such country’. But if so, McCaul argued, the statute must apply to ‘an *Englishman*, as distinguished from a British subject, “country” must refer to “England, not the British Empire” and “sovereign” must mean “the *English* sovereign—not the King, Rex et Imperator, Sovereign of the British Empire”’.^115^ Otherwise, Dicey’s rules would ‘be wide enough in terms to give effective jurisdiction to the courts of any colony (i.e. “foreign” country) over any British subject, wherever resident, since they are all “*subjects of the sovereign of such country*”’.^116^

Furthermore, in a series of legal opinions issued in 1905 and 1906, Robert Randolph Garran, the Law Officer of the Commonwealth of Australia, convinced the Commonwealth government that the Merchant Shipping Act, which was understood to offer an Empire-wide code for shipping, in fact did not extend to the colonies.^117^ Since different parts of the empire were separate jurisdictions, the argument went, the Shipping Act could not extend to the colonies by ‘vague “inferences” and “implication”’.^118^ The UK had a right to legislate with extraterritorial effect to cover the colonies only if expressly stated, and the colonies had a similar right. The mutual recognition of territorial effects of legislation would be settled by ‘comity’.^119^ In 1908, Arthur Berriedale Keith understood Garran’s intervention as indicative

of the great difficulties of the present Imperial constitution, when no federal authority exists, and the Dominions have begun to question the authority of the Imperial Parliament to pass enactments applicable to the Empire as a whole, for that in effect is the spirit which produces the ingenious but unconvincing attempt of the Law Officer of the Commonwealth to prove that the Imperial Shipping Code has scarcely anything to do with the Colonies.^120^

## 4. The View from the Metropole

In hindsight, one might expect to find a strong reaction against these inroads to independence made by the dominions via private international law. But that would misunderstand the imperial context which created and fostered this ambivalence about the nature of extraterritorial authority. First, the reaction of the metropole depended on the issues involved and the particular economic interests of the UK. For example, at the 1911 Imperial Conference, the authority of the dominions to pass legislation with extraterritorial effect was discussed with respect to shipping both as a race question and as an economic question. Dominions were eager to take responsibility for regulating shipping not only for their registered ships and the trade in their continental waters, but also for foreign (including UK) ships passing through dominion ports. More specifically, however, they were eager to ensure that ‘the crews should be exclusively white’^121^ and that they be allowed to impose a ‘prohibitive’ tax on steamship operators bringing in ‘Lascars’ or ‘Asiatics’ even if they were ‘internationally’ British subjects.^122^ They wanted to ensure that their labour laws could extend to UK and foreign ships employing people of colour and entering the dominion territorial waters so as to protect ‘white officers, white engineers and white crews’.^123^ UK representatives and the UK Board of Trade addressed the race question only as a gloss.^124^ This was not entirely new, as a couple of years previously the Privy Council had recognised the authority of the dominions to expel ‘aliens’, even if they were British subjects domiciled in other parts of the empire and even if deportation laws had an extraterritorial effect.^125^ Furthermore, the Chief Justice of New Zealand decided in *In re Award of Wellington Cooks’ and Stewards’ Union* that labour laws could apply with extraterritorial effect even if they were meant to discourage the employment of people of colour.^126^ After all, in private correspondence with Dicey, Keith admitted that the imperial government had long taken the position that it will not interfere with the dominions’ assertion of extraterritorial authority to protect ‘the purity of their race as long as they do not mention Chinese and instead refer to Asiatics’. It would ‘not interfere with immigration legislation however clearly it may be intended to exclude Asiatics and other British subjects’.^127^

There was in turn a strong reaction to the dominions’ attempt to regulate shipping generally, because this assertion of extraterritorial authority impacted directly Britain’s economic interests. From an economic point of view, the fear was that if each dominion decided what restrictions to impose on foreign ships entering their ports, foreign powers would retaliate not against ‘the comparatively small shipping registered in the Dominions, but the enormous shipping of the United Kingdom itself’.^128^ In matters of race, the metropole was willing to accept the dominions’ attempt to gain control, but not in matters that were affecting the metropole’s economic interests. The concepts of ‘territorially independent units’ and ‘local citizenship’ which had allowed the dominions to achieve a higher level of autonomy were the same ones allowing them to achieve a white race policy.

Second, the attempts by the dominions in the first decades of the 20th century were particularly acute, but they were relying on a much older legacy of framing imperial authority. Dominions’ attempts to portray a divisible empire would have appeared especially threatening at a time when it seemed most difficult to keep the empire together. But this simply capitalised on a long history of the metropole’s own making. For the better part of the 19th century, private international law ensured a division of authority which allowed the metropole to portray deference to the autonomy of its different colonies while shielding itself from responsibility.^129^

In 1861, in *Re Holmes* the court held that where land in a colony is vested in the Queen by a Colonial Act for the public purposes of the colony, the 1860 Petitions of Right Act did not give jurisdiction to the Court of Chancery to entertain proceedings against the Crown as a trustee of such land.^130^ The case was a perfect example of the difficulty of conceptualising the relationship between the metropole and the colonies at the intersection of private international law and imperial constitutional law. The Solicitor General argued both that the Crown held the land not as a trustee but ‘in capacity as the Sovereign of Canada’ and that the land is situated in Canada ‘and for all judicial purposes that is a foreign country’. The court found a broader argument to dismiss the case—the Queen, while physically present in England, ‘as the holder of Canadian land for the public purposes of Canada, should be considered as present in Canada’.^131^ But it was unclear where such an argument was supposed to fit, since there was no statutory or common law jurisdictional basis for bringing the claim against the Crown in Canada. By provincialising its own authority, the court in effect remitted the case to a non-existent forum in ‘a foreign country’ part of the British Empire. Fourteen years later, in part relying on *Re Holmes*, the Privy Council decided that the plaintiffs, representing certain creditors of the King of Oudh (one of the Indian princely states), could not bring a claim to enforce a charge against the revenues of Oudh against the Secretary of State for India because the property is located in a foreign country and the Secretary of State is ‘also in India’ even if he is also in England.^132^ This early version of a colonial *forum non conveniens* analysis was applied despite a statute purporting to give plaintiffs a choice between suing the Secretary of State in England or in India. Furthermore, the plaintiff, like many colonial subjects, was resorting to British courts after jurisdiction was declined in colonial courts in India, so the fiction that the Secretary of State was ‘also in India’ amounted to an utter denial of justice under the cover of an enlightened provincialising of its own authority and respect for alterity—after all, said the court, ‘there are courts of law and equity for every part of India’.^133^

By 1893, when the House of Lords decided *The British South Africa Company v The Companhia de Moçambique*, provincialising metropolitan authority especially with respect to land in different parts of the empire solidified into a venerable doctrine of private international law.^134^ The court decided that it had no jurisdiction to entertain an action to recover damages for a trespass to land by the South Africa Company (chartered by the Crown to get as large a share in the scramble for Africa as possible) on land claimed by the Portuguese chartered company operating in Mozambique.

Perhaps the most important case in which England condoned, and in fact conceptualised, the provincialisation of its own authority was *Philips v Eyre*,^135^ cited and relied on in the Mozambique decision. *Philips v Eyre* established the ‘double actionality rule’ for transnational torts, according to which a tort committed abroad is actionable in England only if it is actionable as a tort under English law and it is not justifiable by the law of the place where it was committed.^136^ The case involved tort claims brought by two British subjects residing in Jamaica for the injuries they sustained during the brutal repression of the Morant Bay rebellion by Edward Eyre, the governor of Jamaica at the time. According to an indemnity law passed retrospectively by the governor with the assent of the Crown in council, the governor could not be held liable in Jamaica for acts taken during the quashing of the rebellion. The question was whether the indemnity act also ruled out a claim in English courts. In reaching the decision that the indemnity law precluded any successful claim in England, the Exchequer Chamber combined insights from private international law and imperial constitutional law in puzzling ways. On the one hand, it incorporated and solidified^137^ the imperial constitutional law proposition that local legislative assemblies in the colonies were ‘subordinate authorities, but supreme within the limits of the colony for the government of the inhabitants’.^138^ At the same time, it ruled explicitly for the first time in English courts that *comitas gentium* was to be extended to the colonies, with ample and sustained references to colonies as foreign countries with their own subjects. The court decided to reject the claim. Otherwise

the extraordinary anomaly would arise, that an *inhabitant of a colony*, or it might be of a *foreign country*, owing allegiance to its law, and whose rights arise from and are determined by such law, would be able to enforce rights which, by the law of the country to which *he more immediately belonged* have been taken away from him by a law binding upon him.^139^

This blend of imperial constitutional law and private international law was meant to address three arguments presented on behalf of the claimants. First, extending comity to the laws of Jamaica as a foreign country with its own ‘inhabitants’ could refute the claimant’s argument that the indemnity had the effect of taking away the rights of a British subject under English law.^140^ That would have been contrary to the holding in *Calvin’s Case*.^141^ But if Jamaica was akin to a foreign country, the right claimed was not ‘created’ by English law and then taken away by the law of Jamaica: it could only be created or denied in the first place by the law of the country in which the events took place, even if in this case it were a colony of the British Empire.^142^ Furthermore, it was not entirely accurate to assume that rights were taken away from ‘British subjects’ because the plaintiffs ‘more immediately belonged’ to a particular ‘territorial unit’ of the empire, namely Jamaica. The notion of British subjecthood remained rather formal; for all intents and purposes, Jamaica had authority to vest rights for its own subjects. Second and relatedly, in enacting the indemnity law, Jamaica was allegedly not, contrary to the argument of the counsel for the plaintiff, purporting to *exercise* extraterritorial authority; rather, it was creating, modifying or refusing rights for its own ‘inhabitants’ in its own territory. The exercise of authority was *recognised* abroad—in England—via private international law.^143^

There was, however, a final hurdle. Under principles of private international law, a foreign law that ran counter to the fundamental principles of morality of the forum could be rejected under the ‘public policy exception’. But this seemed implausible, since the indemnity law in Jamaica had received royal assent.^144^ From this vantage point, it was hard to maintain that the indemnity law was entirely foreign and so the private international law public policy exception retreated for its imperial constitutional law variant—the repugnancy rule amended (perhaps in part because of the Jamaica controversy itself)^145^ through the 1865 Colonial Laws Validity Act.^146^ According to this amended version of the repugnancy rule, the indemnity law could be rejected only if it violated an imperial regulation that extended specifically to Jamaica. It was not enough for it to violate fundamental principles of English law.

## 5. Conclusion: Postcolonial Lessons

Throughout the 19th century, private international law doctrine developed at a porous meeting point with imperial constitutional law. From one angle, the two fields could develop sufficiently different normativities so that they appeared to offer alternative arguments and create a blend that would have been unavailable in either of them in isolation. In *Phillips v Eyre*, private international law could construct the Jamaican indemnity rule as foreign law, and imperial constitutional law could impose a different threshold for its recognition via the repugnancy rule. In *The British South Africa Company v Moçambique*, private international law could conceptualise the claim to damages for trespass as a ‘local’ (as opposed to a transitory) action, thus barring the English court’s jurisdiction; meanwhile, imperial constitutional law was left to determine whether the acquiescence of the native chiefs to the British corporation (as opposed to the Portuguese) taking possession of the disputed lands was an exercise of sovereignty.^147^ And in *Re Homes*, one could couple the political indivisibility of the Crown under imperial constitutional law with the divisibility of legislative and adjudicatory jurisdiction under private international law so that lawsuits against the Crown could be pushed to the peripheries of the empire.

By the start of the 20th century, this narrative had become entrenched in the legal and political discourse in the dominions in their attempt to secure autonomy from the metropole and a freehand in racial policies. By the mid-1920s, it would have been hard to trace precisely which argument about imperial authority had been crafted in each field. For example, in 1927, John Salmond took stock of the charge that different parts of the British Empire ‘have no valid claim to the name of state’. He considered the objection ‘unfounded’ because ‘a territorial division of a state can be a state … if it fulfils the essential functions of one’. That, in turn, depended ‘on the extent of the autonomy or independent activity which is permitted to it by the constitution’.^148^ But if one had any doubt, one could fall back on Dicey’s use in his conflict of laws digest of the term ‘country’ ‘to denote the territorial area within which a system of territorial law is in force’.^149^ Both perspectives were equally available and interchangeable.

After the enactment of the Statute of Westminster, much of the history of private international law’s contribution to the framing of imperial authority was lost. In 1938, RTE Latham, Dicey’s intellectual opponent on the question of the supremacy of the British Parliament in the British Empire, reviewed Horace Emerson Read’s book on the recognition and enforcement of foreign judgments in the common law countries of the British Commonwealth. For its clarification that the British Empire was made up of separate states *qua* ‘legal districts’, he recommended the book as ‘good material for legal philosophers and salutary reading for naïve Austinian monists’—clearly a snub at Dicey’s theory of the supremacy of the British Parliament.^150^ Yet, as we have seen, Read’s book was in fact a judicious application of Dicey’s other theory—that of statehood under private international law. The prevalence of private international law arguments in the lead-up to the Statute of Westminster got lost in the historical memory of British constitutional law as well as private international law.

But legal scholarship should tap back into this history, because it allows us to make sense of the imperial continuities in both private international law and constitutional law, as well as at their meeting point. For example, private international law scholars have always decried the continuous expansion of the bases for English courts to take jurisdiction over private international law cases that have only a tenuous connection to England.^151^ Yet, the origins of that expansion are explained by and rooted in the colonial context outlined in this article. Given that different parts of the UK and of the British Empire were conceptualised as ‘foreign countries’, the only way to ensure that English courts could exert jurisdiction over private law disputes scattered across the realm was to expand the range of connecting factors that allowed English courts to assume jurisdiction over ‘transnational’ legal matters. This led to massive revolts from Scotland and Ireland, portraying the extension of jurisdiction as a violation of the Acts of Union.^152^ Scottish and Irish lawyers understood what we have now forgotten—that a change in private international law rules can be motivated by and have massive repercussions for broader constitutional law and devolution questions.

The imperial history of private international law also allows us to trace continuities in contemporary cases, which often rely explicitly on the historical cases discussed in this article. For example, in 2018, a claim brought by 34 individuals against the UK government for alleged assaults, beating, rape and other acts of violence against them between 1956 and 1958 during the uprising against British colonial rule in Cyprus was rejected. Relying on *Phillips v Eyre*, the court applied the law in Cyprus as it stood in 1956 and refused to invoke the public policy exception on the premise that

it makes no difference whether the relevant territory is a colony (whose laws are made by the Queen in Council) or a colony or dominion with its home-grown legislature. The law of the colony in Cyprus is as much a foreign law … as the law of France or the United States.^153^

Likewise, in 2024, a claim was brought in the English courts against the governor of Diego Garcia by Sri Lankan refugees who had been rescued on the high seas and brought to the Chagos Islands.^154^ They were seeking injunctive relief preventing their return from Rwanda (where they had been sent temporarily for medical treatment) to the Chagos Islands, where they alleged inhumane living conditions. The familiar blend outlined in this article between private and public, constitutional law and conflict of laws reasoning, was employed by the court. Under public law, the British Indian Ocean Territory (BIOT) was viewed as a separate jurisdiction from England, but not a sovereign state. Under private law, the issue of designating the BIOT as a separate jurisdiction could be partly avoided^155^ because the governor could be sued in English courts in his individual capacity, as he is domiciled in England. But the blend produced awkward consequences. The court considered it a ‘live issue, whether the proceedings, as a whole, are properly characterised as public law or private law proceedings’,^156^ making it ultimately uncomfortable to recognise jurisdiction against the governor directly under private law, while determining the merits of the injunction and awarding relief based on public law consideration. After all, the court emphasised that the governor, acting on behalf of the BIOT, had no authority to grant the refugees safe passage to the UK, which is a separate jurisdiction.^157^

The puzzle of designating different colonies as extensions of the metropole or separate territorial ‘legal units’ also resurfaced in *R v Secretary of State for Foreign and Commonwealth Affairs (Appellant)*, which also cited and relied on *Phillips v Eyre* and various other cases, which were once squarely at the intersection between private international law and imperial constitutional law.^158^ The House of Lords had to decide whether an order of the Crown in council forcibly deporting Chagossians from the BIOT was *ultra vires*. The majority ruled that it was not. Two aspects were particularly important in answering the question: first, whether the law forcibly deporting Chagossians could be said to contravene fundamental principles of English law; and second, whether the law was irrational because it failed to consider the interests of the Chagossians.

On the first aspect, the historical blend of designating colonies as both foreign and inherent parts of the empire was applied to BIOT.^159^ The BIOT was a separate ‘legal unit’, with its own law. But its laws could only be disapplied if they contravened specific legislation extending to the BIOT as per the Colonial Laws Validity Act of 1865 applied in *Phillips v Eyre*. Neither human rights law nor the Magna Carta applied there. According to Lord Hoffmann, this can be explained by ‘the significant words … [of] the law of the land’ in the Magna Carta, clarifying that this is the law of England, but not the law of the colony.^160^

However, on the second point, of whether or not the law was irrational because it failed to consider the interests of the Chagossians, the court portrayed an indivisible realm. Allegedly, legislating for the BIOT did not imply a restriction to focus exclusively or even primarily on the interests of the inhabitants of the island rather than on the interests of the whole realm.^161^ Viewed from the vantage point of the shared imperial history of private international law and constitutional law, it is clear that the case brings back to light and exacerbates the paradox of claiming both an indivisible realm and a division between legal units of the post-imperial UK.

At a broader analytical level, acknowledging the imperial interplay between private international law and constitutional law creates an opening for each field. Private international law should take stock of its own very particular political theory of the state and start tracing the impact it has had in different historical and geopolitical contexts. Contemporary private international law scholars imagine the field as a repertoire that is only necessary and accessible once a fully formed legal system is in existence. Yet, its imperial history shows that the field often plays a significant role in conceptualising legal systems from the ground up. This was not limited to the white settler dominions. In 1926, Arthur Berriedale Keith decried that scholars were not paying enough attention to the way in which Indian princely states or the unfederated state of Kelantan were being ‘treated as independent sovereign states by private international law’, while they were ‘denied by the British Government to be within the purview of public international law’.^162^ In turn, those cases were used by the Foreign Office to explain why Gulf State protectorates could be seen as sovereign states in private international law even though they were not recognised as such in either UK constitutional law or public international law.^163^

In private correspondence with Keith, Dicey often decried that the imperial structure had not managed to grant sufficient autonomy to the dominions, while securing ‘perfect equality’ for all British subjects. What if, he asked, ‘New Zealand introduced a system of trial which in English opinion were unfair to the Maoris or, to take an extreme case, legalized in some instances the use of torture?’ In deciding to recognise the authority of a dominion in a particular case, was not England simultaneously making a determination about ‘British ideas of morality?’^164^ Much the same question can be asked today in cases brought against UK foreign service personnel implicated in the torture of detainees in black sites around the world,^165^ against British corporations alleged to have condoned forced labour to benefit business operations abroad^166^ or by asylum seekers demanding safe passage from the Chagos Islands to the UK.^167^

The lessons are equally significant for British constitutional law theory today. Constitutional law scholars are struggling to define the nature of the UK constitution, somewhere in between a unitary and a federal model.^168^ But from the recent cases on British Overseas Territories, it seems more likely that the imperial ambivalence about describing an undivided realm for some purposes and a divided legal pluralist scene for others continues to describe the British constitutional law landscape. Some are tapping into imperial history to trace different ways of conceptualising legislative and political independence for parts of the British Empire, to then compare and contrast them with contemporary legislation and contemporary concerns of devolved governments. Historically ‘thinner’ versions of autonomy and sovereignty are contrasted with contemporary ‘thicker’ versions.^169^ While extremely valuable, that history is incomplete and the lessons to be learned are only partial, without tracing the way in which these notions of sovereignty and statehood were made possible through the constant blend of constitutional and private international law doctrines and principles.

## Footnotes

1 A Wood Renton, ‘A Century of Colonial Juridical Policy (Continued)’ (1932) 44 Jur Rev 221, 221.

2 Lauren Benton, *A Search for Sovereignty: Law and Geography in European Empires, 1400–1900* (CUP 2009); Lisa Ford, *Settler Sovereignty. Jurisdiction and Indigenous People in America and Australia 1788–1836* (Harvard UP 2011); Lauren Benton and Lisa Ford, *Rage for Order. The British Empire and the Origins of International Law (1800–1850)* (Harvard UP 2018); Mitra Sharafi, *Law and Identity in Colonial South Asia: Parsi Legal Culture, 1772–1947* (CUP 2014); Will Hanley, *Identifying with Nationality. Europeans, Ottomans and Egyptians in Alexandria* (Columbia UP 2017); Nurfadzilah Yahaya, *Fluid Jurisdictions: Colonial Law and Arabs in Southeast Asia* (Cornell UP 2020).

3 Paul F Scott, ‘Special Issue: The Constitutional Legacies of Empire’ (2020) 71 NILQ 99; Peter C Oliver, *The Constitution of Independence. The Development of Constitutional Theory in Australia, Canada, and New Zealand* (OUP 2005); David Dyzenhaus and Thomas M Poole, ‘The Old Commonwealth Model of Constitutionalism’ (LSE 2023) LSE Legal Studies Working Paper No 11/2023 <https://papers.ssrn.com/sol3/papers.cfm?abstract_id=4414387>.

4 Thibault Guilluy, ‘Visions of Constitutionalism: The Implementation of Representative Institutions in the United Kingdom’ in Kelly L Grotke and Markus J. Prutsch (eds), *Constitutionalism, Legitimacy and Power: Nineteenth-Century Experiences* (OUP 2014).

5 H Kumarasingham, ‘Constitution and Empire’ in Peter Cane and H Kumarasingham (eds), *The Cambridge Constitutional History of the United Kingdom*, vol 2 (CUP 2023).

6 But see Nicole Stybnarova, ‘The (Neo-)Imperial Economic Functionality of the International Regulation of Marriage’ Am J Comp L (forthcoming); Sandrine Brachotte, ‘Pour une approche décoloniale du droit international privé’ (2023) 2 Revue critique de droit international privé 297; Susanne Lilian Göss, ‘Fortsetzung des Kolonialismus im internationalen Recht der Gegewart? Grundlagen, Strukturen, Methoden aus der Perspektive des IPR (2024) 52 Berichte der Deutschen Gesellschaft für internationales Recht 45; Ralf Michaels, ‘Internationales Privatrecht und der globale Süden’ (2025) 4 Praxis des Internationalen Privat- und Verfahrensrechts 377.

7 Jacco Bomhoff, ‘The Constitution of the Conflict of Laws’ in Horatia Muir Watt and Diego P Fernández Arroyo, *Private International Law and Global Governance* (OUP 2014).

8 Renton (n 1) 221.

9
*BAA, BAB, BAC, BAD, BAE v Commissioner of the British Indian Ocean Territory Administration (Paul Candler), The Secretary of State for the Foreign, Commonwealth and Development Office, Secretary of State for Defence* [2023] EWHC 767 (KB). The cases referenced in this article predate the 2024 agreement between the UK and the Republic of Mauritius concerning the Chagos Archipelago.

10
*R v Secretary of State for Foreign and Commonwealth Affairs* [2008] UKHL 61, paras 43 and 65.

11 ibid 49–52.

12 Ewan Smith, ‘Devolution after Empire’ OJLS (forthcoming).

13 ‘Legal pluralism’ in the context of this article refers simply to the acceptance, under private international law, of multiple ‘state’ legislative and adjudicatory authorities throughout the empire, in contrast to the unitary legislative framework portrayed by classical accounts of imperial constitutional law premised on the supremacy of the Westminster Parliament. This is therefore a weaker form of pluralism, different from both the ‘juristic’ and the ‘social’ type described in SE Merry, ‘Legal Pluralism’ (1988) 22 L & Soc’y Rev 869.

14 A helpful way to trace the development of private international law in this way is to compare the editions of Burge’s *Commentaries on Colonial and Foreign Laws* published in 1838 and 1841 with the 1908 edition.

15 This framing is inspired by the metaphor of the double-facing constitution developed in Jacco Bomhoff, David Dyzenhaus and Thomas Poole (eds), *The Double-Facing Constitution* (CUP 2020).

16 Charles James Tarring, *Chapters on the Law Relating to the Colonies to Which are Appended Topical Indexes of Cases Decided in the Privy Council on Appeal from the Colonies, Channel Islands and the Isle of Man, and of Cases Relating to the Colonies Decided in the English Courts* (3rd edn, Stevens & Haynes 1906) 60–4.

17 ibid 64 (‘the power of the crown to negative or veto the Bills of colonial legislatures is virtually the right of the Imperial Parliament to limit colonial legislative independence, and is frequently exercised’.).

18 See Alexander Wood Renton and George Grenville Phillimore (eds), *Burge’s Commentaries on Colonial and Foreign Laws Generally and in Conflict with Each Other and with the Law of England, vol 1* (Sweet & Maxwell 1907); Michael Crowder, ‘Indirect Rule: French and British Style’ (1964) 34 Africa 197.

19 Tarring (n 16) 4–6.

20
*Campbell v Hall* (1774) 1 Cowp 204, 209; 98 ER 1045, 1047 (KB).

21 See eg the Gold Coast Ordinance No 4 of 1876, stipulating that English law in force on 24 January 1874 is to be observed so far as the limits of local jurisdiction and circumstances permit; but native laws are to be respected as far as possible.

22 Tarring (n 16) 7.

23 James S Read, ‘Customary Law Under Colonial Rule’ in HF Morris and James S Read (eds), *Indirect Rule and the Search for Justice* (Clarendon Press 1972) 167.

24 For New Zealand see eg Tarring (n 16) 6.

25 For the context of the Straits Settlement, see Yahaya (n 2).

26 For example, Jamaica was portrayed as a settled colony in *Campbell v Hall* (1774) 1 Cowp 204, 98 ER 1045 and as a conquered colony in *Beaumont v Barrett* (1836) 1 Moo PC 75, 12 ER 733.

27 See Peter C Oliver, ‘“Dominion Status”: History, Framework and Context’ (2019) 17 International Journal of Law in Context 1173; Arthur Berriedale Keith, *Responsible Government in the Dominions*, vol 1 (Clarendon Press 1912) 355–423.

28 DB Swinfen, ‘The Genesis of the Colonial Laws Validity Act’ (1967) Jur Rev 29; Enid Campbell, ‘Colonial Legislation and the Laws of England’ (1965) 2 U Tas L Rev 148.

29 See Tasim Olawale Elias, *English Colonial Law. A Comparative Study of the Interpretation Between English and Local Laws in British Dependencies* (Stevens & Sons 1962) 53. See also Thomas Baty, ‘The Structure of the Empire’ (1930) 12 J Comp Leg 157; SD Sharma, ‘Applicability of the Doctrine of Extra-Territoriality to Legislation by the Indian Legislature’ (1946) 28 J Comp Leg 91.

30 See Baty, ‘The Structure of the Empire’ (n 29).

31 Albert Venn Dicey, ‘Ireland and Victoria’ (1886) 49 The Contemporary Review 169, 169.

32 Tarring (n 16) 58.

33 See Keith, *Responsible Government* (n 27) 355–72; RW Shannon, ‘Delegated Legislation’ (1928) 4 Can Bar Rev 245.

34 Arthur Berriedale Keith, *The Sovereignty of the British Dominions* (Macmillan 1929).

35 Thomas Baty, ‘Sovereign Colonies’ (1921) 34 Harv L Rev 837, 850.

36 See *G Gibson & Co v Gibson* [1913] 3 KB 379. Thomas Baty, ‘The History of Canadian Nationality’ (1936) 18 J Comp Leg 195.

37 John Salmond, ‘Territorial Waters’ (1918) 34 LQR 235. See also letter of Arthur Berriedale Keith to AV Dicey, 6 August 1906 in Ridgway F Shinn Jr and Richard A Cosgrove, *Constitutional Reflections. The Correspondence of Albert Venn Dicey and Arthur Berriedale Keith* (University Press of America 1996) 5: the courts ‘might differentiate between a Colony and a foreign country, but of course for years we have always held that a Colony’s legislation was purely territorial and confined within the three miles’ limit’.

38 Frank Anglin, ‘Extra-Territorial Criminal Legislation of Canada’ (1900) 19 CLT 1; 19 CLT 38, 44.

39 See T Baty, ‘Sovereign Colonies’ (1921) 34 Harv L Rev 837, 853.

40 ibid 846.

41 See Roxana Banu, *Nineteenth Century Perspectives on Private International Law* (OUP 2018).

42 See William Burge, *Commentaries on Colonial and Foreign Laws* (Saunders and Benning, 1838).

43 See Shinn and Cosgrove (n 37) ch 1.

44 Mark Walters, *AV Dicey and the Common Law Constitutional Tradition: A Legal Turn of Mind* (CUP 2020).

45 But see Roxana Banu, ‘Reading Dicey’s Private International Law in the Penumbra: Empire and Gender’ in Andrew Dickinson, Timothy Endicott and Ernst Wolfgang (eds), *Dicey + 100: Albert Venn Dicey: A Centennial Commemoration* (Intersentia 2024); Ugljesa Grusic, *Torts in UK Foreign Relations* (OUP 2023).

46 See Shinn and Cosgrove (n 37).

47 Dylan Lino, ‘The Rule of Law and the Rule of Empire: AV Dicey in Imperial Context’ (2018) 81 MLR 739.

48 AV Dicey, *The Law of Domicil as a Branch of the Law of England, Stated in the Form of Rules* (Stevens & Sons 1879) 30ff.

49 AV Dicey, *A Digest of the Law of England with Respect to the Conflict of Laws* (Stevens & Sons 1896) 67ff.

50 Dicey, *The Law of Domicil* (n 48) 32.

51 ibid.

52 ibid 31 note e.

53 ibid.

54 ibid 32. Note that Dicey’s notion of a ‘territorial unit’ provided a much lower threshold for statehood under private international law than that provided in 1933 in the Montevideo treaty under public international law. Article 1 of the Convention on the Rights and Duties of States, adopted on 26 December 1933, provides for: a permanent population; a defined territory; a government; and the capacity to enter into relations with other states. The population of the dominion was hard to conceptualise, and one had to rely on private international law notions of domicile for this purpose. The capacity of the government to enter into relations with other states was limited or denied entirely. Thus, the framework Dicey offered moved significantly beyond the recognition of ‘de facto’ states discussed by Lucia Leontiev. See Lucia Leontiev, ‘Objects and Subjects of Extraterritoriality: The Case of De Facto States’ in Hannah L Buxbaum and Thibaut Fleury Graff (eds), *Extraterritoriality* (Brill 2022) 315.

55 Dicey, *The Law of Domicil* (n 48) 33.

56 Since WWI, several accounts have contemplated the relevance of private international law’s own theory of the state in different contexts. See Amos Shapira and Hala Tabory, *New Political Entities in Public and Private International Law with Special Reference to the Palestinian Entity* (Kluwer 1999); Alexander Tunk and others, *Non-Recognised States in the Post-Soviet Region under International Trade Law, Private International Law and Civil Procedure* (Springer 2024); Jurgen Basedow, ‘Non-recognised States in Private International Law’ (2019) 20 Yearbook of Private International Law 1.

57 Baty, ‘Sovereign Colonies’ (n 39) 678.

58 See Dicey’s comments on the case *In re Orr Ewing*, 1882, 22 Ch D (CA) 456, 464 and 465, given Jessell MR’s view of the word ‘foreign’ as applied to Scotland in Dicey, *A Digest* (n 49).

59 Antoine Pillet, *Recherches sur les droits fondamentaux des États dans l’ordre des rapports internationaux et sur la solution des conflits qu’ils font naître* (Pedone 1899).

60 For a discussion of the references in Dicey’s digest to unequal civilisation, see Antoine Pillet, ‘Sur un point peu aperçu de la doctrine de Dicey’ (1923) 4 Revue de Droit International et de la Legislation Comparée 345, 355.

61 PE Nygh, ‘The Territorial Origin of English Private International Law’ (1964) 2 U Tas L Rev 28; Alexander N Sack, ‘Conflict of Laws in the History of English Law’ (1937) 3 Law: A Century of Progress 344.

62 See Janet McLean, *Searching for the State in British Legal Thought. Competing Conceptions of the Public Sphere* (CUP 2012).

63 Emerson Read traced the notion of territoriality that informed the view that colonies are akin to foreign states to the imperial doctrine of ex-territoriality, rather than earlier 17th-century Dutch theories. See HE Read, *Recognition and Enforcement of Foreign Judgments in the Common Law Units of the British Commonwealth* (Harvard UP 1938) 3–6. In reviewing Read’s book on the recognition and enforcement of foreign judgments in the common law units of the British Commonwealth, American scholar Hessel Yntema noted that the context of the British Empire offered a ‘startling discovery’ that the concept of territoriality and the vested rights theory were inoperable and ‘rest upon a natural law theory about law, which is no longer convincing’. See Hessel Yntema (1940) 49 Yale LJ 1134, 1135–6 and 1139.

64 Read (n 63) 61.

65 ibid 5 (emphases added).

66 RA Reid, ‘Company Incorporation Jurisdiction in Canada under the B.N.A. Act 1867’ (1912) 32 CLT 749.

67 Harrison Moore, ‘The Federations and Suits Between Governments’ (1935) 17 J Comp Leg 163; Harrison Moore, ‘Suits Between States Within the British Empire’ (1925) 7 J Comp Leg 155.

68 John Salmond, ‘Citizenship and Allegiance’ (1902) 18 LQR 49.

69 Domicile had a very particular meaning in private international law, so that a domicile of origin would be lost only on the establishment of a domicile of choice based on an intention to reside in the new place permanently or indefinitely. See *Bruce v Bruce* (1790) 2 Bos & Pul 229; 169 ER 908. This notion posed significant difficulties in the imperial context, where many Englishmen and Scots resided in the empire for very long periods without being considered domiciled there. See Arthur Berriedale Keith, ‘Some Problems in Conflict of Laws’ (1935) 16 Bell Yard: Journal of the Law Society’s School of Law 4. Therefore, those, like John Salmond, who argued for local citizenship based on domicile avoided providing a clear definition of domicile.

70 AH Lefroy, *Canada’s Federal System Being a Treatise on Canadian Constitutional Law under the British North America Act* (Carswell 1913) 103–4.

71 Dicey, ‘Ireland and Victoria’ (n 31) 172.

72 ibid.

73 For example, several dominions were attempting to take jurisdiction to issue a divorce for deserted women who were considered (according to the English doctrine of dependent domicile) domiciled in England based on the husband’s domicile. See letter from Arthur Berriedale Keith to AV Dicey on 22 September 1906 in Shinn and Cosgrove (n 37) 7–14.

74 For an attempt at a public international law argument, see Mr Justice Gwynne, *In Re Criminal Code, 1982 and sections relating to bigamy* (1897) 27 SCR 461, 480.

75 For a philosophical account of the constitutive nature of the recognition of authority, see Ralf Michaels, ‘Law and Recognition—Towards a Relational Concept of Law’ in Nicole Roughan and Andrew Halpin (eds), *In Pursuit of Pluralist Jurisprudence* (CUP 2017).

76 For a broad historical account, see DP O’Connell, ‘The Doctrine of Colonial Extraterritorial Legislative Incompetence’ (1959) 75 LQR 318; Keith, *Responsible Government* (n 27).

77 Herbert Smith, ‘Extra-territorial Legislation’ (1923) 1 Can Bar Rev 338, 346: ‘The theory that Dominion legislatures are subject to some vague territorial limitation from which the British Parliament is free is in reality part of the old idea, never precisely defined, that they are in some sense imperfect legislatures of an inferior type, a notion which is wholly at variance with the principle laid down in 1883 in the celebrated case of Hodge v The Queen.’

78
*Macleod v New South Wales* [1891] AC 455.

79 Even then, however, it would have been unclear whether the British subjects to which the Canadian statute would have referred had to have a connection to Canada in particular and, if so, whether that amounted to domicile or a long residence. See eg the discussion in *Attorney-General for Canada v Cain and Gilhula* [1906] AC 542; Keith, *Responsible Government* (n 27).

80
*Macleod* (n 78).

81
*Keyes v Keyes* [1921] P 204. Before the Indian and Colonial Divorce Jurisdiction Act 1926, this was the case in India as well. Even after the passing of the Act, it was unclear whether domicile or residence was the proper connecting factor for jurisdiction in divorce matters. For a discussion, see Priyasha Saksena, ‘Limping Marriages: Race, Class, and the Rise of Domicile-Based Divorce Jurisdiction in the British Empire’ (2023) 63 Am J Legal Hist 36.

82
*Isaacs & Sons v Cook* (1925) 2 KB 391.

83
*London and South American Investment Trust Limited v British Tobacco Co (Australia)* [1927] 1 Ch 107. See also the discussion in Berriedale Keith, ‘Das Verhältnis des Statute of Westminster von 1931 zum internationalen Privatrecht’ (1932) 6 Zeitschrift fuer ausländisches und internationalen Privatrechts 301, 305–6, pointing out that this decision had significant implications for commercial activities within the British Empire.

84 See Manley O Hudson, ‘Notes on the Statute of Westminster’ (1931) 46 Harv L Rev 261, 274 fn 61 (citing the report of the 1929 Conference & Lord Sankey in HL Deb, 26 November 1931, vol 83, col 182).

85 Section 1 of the Statute provided: ‘In this act the expression “Dominion” means any of the following Dominions, that is to say, the Dominion of Canada, the Commonwealth of Australia, the Dominion of New Zealand, the Union of South Africa, the Irish Free State and Newfoundland’.

86 For an account of the debates on the geographical reach of the term ‘British Commonwealth of nations’, see Hudson (n 84) 269. For a discussion of the different treatment given to dominions as opposed to colonies, see Elias (n 29).

87 For a discussion of the impact of the early 20th-century debates on extraterritoriality in Canada in the later-formed dominion of the Irish Free State, see Thomas Mohr, ‘The Foundations of Irish Extraterritorial Limitation’ (2005) 40 IJ 86. For India, see Sharma (n 29).

88 On Moore, see James Cotton, ‘Imperialism and Internationalism in Early Australian International Relations: The Contribution of W. Harrison Moore’ (2009) 44 Australian Journal of Political Science 639.

89 W Harrison Moore, ‘The Sources of the Laws of the Colonies’ (1900) 2 Journal of the Society of Comparative Legislation 276.

90 ibid 278.

91 ibid 281.

92 AHF Lefroy, *Canada’s Federal System; Being a Treatise on Canadian Constitutional Law Under the British North America Act* (Carswell 1913) 103.

93 AH Lefroy, *The Law of Legislative Power in Canada* (Toronto Law Book and Publishing Co 1897–98) 328–33.

94 See Lefroy, *Canada’s Federal System* (n 92) 105–6, fn 23.

95 Walter S Scott, *The Canadian Constitution: Historically Explained by Annotated Statutes, Original Documents and Leading Cases* (Carswell 1918).

96 John M Law and Roderick J Wood, ‘A History of the Law Faculty’ (1996) 35 Alta L Rev 1, 7.

97 Scott, *The Canadian Constitution* (n 95) 8–9.

98 ibid 8–14.

99 In addition to editing Dicey’s digest on conflict of laws and being a widely recognised expert in imperial constitutional law, Keith also edited *Wheaton’s Principles on International Law* for two editions (1929 and 1944).

100 Keith, *Responsible Government* (n 27) 394–5.

101 ibid 395.

102 Shinn and Cosgrove (n 37) 5.

103 This is why the Order in Council applying s 20 of the Finance Act of 1894 had to be revoked when applied to the Cape Colony. See A Keith, *Responsible Government* (n 27) 395.

104 Shinn and Cosgrove (n 37) 5.

105 John W Salmond, ‘Limitations of Colonial Legislative Power’ (1917) 33 LQR 117, 121.

106 ibid 121.

107 ibid 122: ‘The very same considerations which have induced the Parliament of the United Kingdom to pass in the interests of that Kingdom laws which relate to matters beyond the territorial limits apply with equal force to the legislation and the interests of the dependencies of the Crown. If it is necessary and permissible for the Parliament of the United Kingdom to pass extra-territorial laws for the regulation of coastal fisheries, or for the effective administration of the customs law, or for the deportation of undesirable aliens, or for the punishment in England of certain crimes committed by British subjects abroad, why is it not equally necessary and permissible for similar measures to be enacted in the colonies for their own protection and in their own interests?’

108 Smith, ‘Extra-territorial Legislation’ (n 77); Herbert Smith, ‘The Legislative Competence of the Dominions’ (1927) 43 LQR 378, 387.

109 Smith, ‘Extra-territorial Legislation’ (n 77) 346. Herbert Smith wrote an article proposing the founding of an imperial school of law which would have placed significant emphasis on the study of conflict of laws: ‘Probably we shall see the day when Dicey’s great work on the Conflict of Laws will also receive the sanction of Parliament. See Herbert Smith, ‘An Imperial School of Law’ [1927] JSPTL 11.

110 Salmond, ‘Limitations’ (n 105) 120.

111 CC McCaul, ‘Some Aspects of the Law of Foreign Judgments with Special Reference to Default Judgments of English and Colonial Courts Inter Se’ (1908) 24 LQR 412, 424–5.

112 Francis Taylor Piggott, *Service Out of the Jurisdiction* (William Clowes & Sons 1892) 201–2.

113 McCaul (n 111) 427 (emphasis as in original).

114 ibid 428.

115 McCaul took all this from Dicey, see McCaul (n 111) 429 and fnn 3 and 4 (emphases as in the original).

116 ibid 429 (emphasis as in the original).

117 See Garran, Opinion Nos 211 (6 February 1905) and 229 (15 January 1906).

118 See Garran, Opinion No 229 (n 117) para 15.

119 Garran, Opinion No 211 (n 117). Note that it was very common for private international law to be referred to as ‘comity’. See FC, ‘Comity of Nations’ (1844) 31 Law Mag Quart Rev Juris 276.

120 Arthur Berriedale Keith, ‘Merchant Shipping Legislation in the Colonies’ (1908) 9 Journal of the Society of Comparative Legislation 202, 221.

121 Minutes of the Imperial Conference (New Zealand) 1911, presented to both houses of the General Assembly by command of His Excellency, 138.

122 ibid 405. Lascars was the pejorative term used to designate sailors or militiamen from the Indian subcontinent, Southeast Asia, the Arab World, British Somaliland or other parts east of the Cape of Good Hope who were employed on European ships.

123 ibid 400.

124 ibid 394ff.

125
*Attorney-General for Canada v Cain and Gilhula* [1906] AC 652

126
*In Re Award of Wellington (Cooks and Stewards’ Union* (1906) 26 NZLR 394.

127 Shinn and Cosgrove (n 37).

128 Keith, ‘Merchant Shipping Legislation’ (n 120) 203.

129 For a discussion of suspending free press rights in the colonies, even if it was understood that this would diverge from fundamental values of English law, see Campbell (n 28) 159.

130
*Re Holmes* (1861) 70 ER 1167.

131 ibid 1172–4.

132
*Doss v Secretary of State for India in Council* (1875) LR 19 Eq 509, 535.

133 ibid 535–6. See also Prabhakar Singh, ‘Indian Princely States and the 19th-Century Transformation of the Law of Nations’ (2020) 11 JIDS 365.

134
*The British South Africa Company v The Companhia de Moçambique and Others* [1893] AC 602.

135
*Phillips v Eyre* (1870) LR 6 QB 1.

136 The Rule was abolished in England by part III of the Private International Law (Miscellaneous Provisions) Act 1995, but it still applies in England, Wales and Northern Ireland to torts committed before 1994 and in defamation claims. It remains in force in various forms in other jurisdictions, such as Hong Kong and Singapore.

137 The case was cited as definitive authority in one of the earliest cases on the extraterritorial competence of dominion legislatures, namely *Ray v Mc’Makin* (1875) 1 VLR(L) 274, 280.

138
*Phillips v Eyre* (1870) (n 135) 20.

139
*Philips v Eyre* (1869) LR 4 QB 225, 242 (emphases added).

140
*Phillips v Eyre* (1870) (n 135) 28–9.

141
*Calvin’s Case* (1608) 7 Co Rep 1a, 77 ER 377.

142
*Philips v Eyre* (1869) (n 139) 238–40.

143 ibid; *Phillips v Eyre* (1870) (n 135) 25.

144
*Philips v Eyre* (1869) (n 139) 243.

145 For a similar view, see John Finnis, ‘Common Law Constraints: Whose Common Good Counts?’ (University of Oxford Faculty of Law, 2008) Legal Studies Research Paper Series, Working Paper No 10/2008, 4 fn 14 <www.cpl.law.cam.ac.uk/sites/www.law.cam.ac.uk/files/images/www.cpl.law.cam.ac.uk/legacy/File/Finnis_J_Paper%281%29.pdf> (accessed 9 February 2026).

146 On the history of the statute, see Swinfen (n 28). For a discussion about parallels between the repugnancy rule and the public policy rule in the context of the colonial government of Africa, see RD Leslie, ‘The Repugnancy Rule in African Law and the Public Policy Rule in Conflict of Laws’ (1977) Acta Juridica 117.

147
*The British South Africa Company and the Companhia de Moçambique and Others* [1893] AC 602, 629–30.

148 John Salmond, *Jurisprudence* (7th edn, Sweet & Maxwell 1924) 158.

149 ibid 89 note (e).

150 RTE Latham, ‘Review Article: Recognition and Enforcement of Foreign Judgments in the Common Law Countries of the British Commonwealth’ (1939) 18(2) Foreign Affairs 278.

151 See eg Alex Mills, ‘Exorbitant Jurisdiction and the Common Law’ in Jonathan Harris and Campbell McLachlan (eds), *Essays in International Litigation for Lord Collins* (OUP 2022) 243–64.

152 See National Archives, LCO 2/555, Scottish Objections to Order XI, Rule 1. See also Roxana Banu & Brooke Marshall, ‘Choice of Law and Jurisdiction: Rash and Radical, or Reasoned and Rational’ (draft, on file with author).

153
*Athanasios Sophocleous & Ors v Secretary of State for the Foreign and Commonwealth Office & Secretary of State for Defence* (2018) EWCA Civ 2167, paras 40 and 44.

154
*BAA, BAB, BAC, BAD and BAE v Commissioner of the British Indian Ocean Territory Administration, The Secretary of State for the Foreign, Commonwealth and Development Office, Secretary of State for Defence* [2023] EWHC 767 (KB).

155 I say partly because the court engaged in a condensed *forum non conveniens* analysis which recognises that there is another available forum in Diego Garcia.

156
*BAA, BAB, BAC, BAD and BAE* (n 154) 74.

157 ibid 49–57.

158
*R v Secretary of State for Foreign and Commonwealth Affairs* (n 10) paras 32, 36, 50, 81, 84, 88, 97, 101, 108 and 147.

159 For a reading of the Chagos Island case as a continuation of imperial constitutional practices, see Hakeem O Yusuf and Tanzil Chowdhury, ‘The Persistence of Colonial Constitutionalism in British Overseas Territories’ (2019) 8 GlobCon 157.

160
*R v Secretary of State for Foreign and Commonwealth Affairs* (n 10) paras 43 and 65.

161 Finnis (n 143); *R v Secretary of State for Foreign and Commonwealth Affairs* (n 10) paras 28ff.

162 A Berriedale Keith, ‘Review of *Backward Territories in International Law*’ (1926) 8 J Comp Leg 307, 307.

163
*Boundaries of South-Eastern Arabia and Qatar* (British Library) Asian and African Studies, IOR/L/PS/12/2135.

164 Shinn and Cosgrove (n 37) 100.

165
*Zubaydah v Foreign, Commonwealth and Development Office and Others* [2023] UKSC 50.

166
*Limbu and Others v Dyson Technology Ltd and Others* [2024] EWCA Civ 1564.

167
*The Commissioner for the British Indian Ocean Territory v The King (On the Application of VT & Ors)* [2024] BIOT Ca (Civ) 3. The UK has since granted all but three asylum seekers permission to resettle in the UK.

168 Smith, ‘Devolution after Empire’ (n 12).

169 Anurag Deb, ‘Lessons from the Age of Empire: The UK Internal Market Act as a Rupture in the Understanding of Competence’ (2024) 75 NILQ 106.

